# Dietary supplements for prevention of Alzheimer’s disease: *In vivo* and *in silico* molecular docking studies

**DOI:** 10.22038/ijbms.2024.79960.17320

**Published:** 2025

**Authors:** Doha Abdou Mohamed, Rasha Salah Mohamed, Karem Fouda, Hoda Bakr Mabrok

**Affiliations:** 1 Nutrition and Food Science Department, Food Industries and Nutrition Institute, National Research Centre, Dokki 12622, Cairo, Egypt

**Keywords:** Acetylcholinesterase Alzheimer’s disease Amyloid-β, Dietary supplements, Gene expression, Molecular docking

## Abstract

**Objective(s):**

Alzheimer’s disease (AD) is one of the most common neurodegenerative diseases in people over 65. The present research aimed to investigate the potential of different dietary supplements (DS) in preventing AD in an experimental animal model and *in silico* study.

**Materials and Methods:**

Three DS containing a mixture of wheat-germ oil and black pepper extract/or turmeric extract were prepared. Total phenolic content, HPLC-phenolic profile, phytosterols content, fatty-acids profile, and anti-oxidant activity were evaluated in all DS. The protective effect of the prepared DS was assessed through their impact on cholinergic neurotransmission and the gene expression of GSK3β, APP, and Akt. Oxidative stress and inflammatory markers were evaluated. The inhibition activities against acetylcholinesterase (AChE) and reduction of amyloid-β aggregation of the major phytochemicals present in the studied DS were evaluated using *in silico* molecular docking study.

**Results:**

Molecular docking revealed that rosmarinic acid and β-Sitosterol exhibited the strongest binding affinities for AChE and Amyloid-β, respectively. The results showed that all DS reduced cholinergic neurotransmission, decreased TNF-α as an inflammatory marker, and improved oxidative stress status. All DS down-regulated the expression of GSK3β and APP while significantly up-regulating the expression of the Akt gene.

**Conclusion:**

The present study concluded that all DS enhanced cholinergic neurotransmission, reduced inflammation, and improved oxidative stress status by impacting the expression of GSK3β, Akt, and APP genes. Rosmarinic acid and β-sitosterol showed promising effects for treating AD, according to an *in silico* molecular docking study. The studied dietary supplements were considered promising candidates for the prevention of AD.

## Introduction

Alzheimer’s disease (AD) is one of the most common neurodegenerative diseases that result in cognitive impairment, memory loss (dementia), and behavioral alterations in about 10% of people over 65 years of age, especially women (1). AD is responsible for 60% to 80% of dementia cases (2). In 2050, AD will affect 115.4 million people, and it is one of the most important reasons for death globally (3). Numerous hypotheses attempt to explain AD pathogenesis, such as the amyloid, cholinergic, and tau hypotheses. Additionally, other factors that play a role in AD diseases include oxidative stress, neuroinflammation, head injuries, vascular disease, genetic factors, and environmental factors (4-6). 

AD is characterized by amyloid plaques, neurofibrillary accumulation, cholinergic function impairment, and neural loss tangles in the AD patient’s brain (7). In AD, the main component of amyloid plaques is amyloid β protein (Aβ), which has been considered as the main initiating factor for AD pathological processes. Aβ is generated by sequential cleavages of amyloid precursor protein (APP) by β-secretase and γ-secretase. The imbalance between the synthesis of Aβ and clearance of Aβ leads to aggregation, protein misfolding, and extracellular accumulation, which finally results in amyloid plaque formation (8). The abnormal accumulation of Aβ and amyloid plaques in the cerebral cortex and hippocampus leads to neurotoxicity and causes synapse loss, axon damage, and cognitive impairments (9, 10). Aβ can affect cholinergic neurotransmission by reducing the release of the neurotransmitter acetylcholine, decreasing choline-acetyltransferase activity, and increasing acetylcholinesterase activity (AChE), which blocks signal transmission (11, 12). The interaction between AChE and Aβ can participate in the AD progression amplification (13). 

There are several approaches targeting signaling pathways that may give a potential therapeutic agent to slow the progression of AD. One therapeutic strategy that elevates neural cell function and cognitive function is increasing levels of cholinergic by AChE inhibitors (14). Moreover, developing a therapeutic compound targeting the Aβ pathway and modulating APP cleavage is another approach for AD treatment. One possible signaling pathway targeting the treatment of AD is the glycogen synthase kinase-3 (GSK3β) pathway (15). GSK3β plays a critical role in AD pathology; it is found to be highly expressed in the brains of AD patients. GSK3β activation in the brain of AD patients regulates APP cleavage and promotes Aβ accumulation, tau hyper-phosphorylation, neural dysfunction, and inflammatory molecule production (16). Hence, dietary supplements containing active compounds may play an important role as inhibitors for AChE and GSK3β. So, dietary supplements may be a promising agent for preventing and treating AD.

Plant phytochemicals exhibited many biological activities such as neuroprotective activity, anti-inflammatory, anti-oxidant, anti-diabetic, and cardioprotective effects (17). Several plants and phytochemicals are recommended in traditional practices of medicine to boost cognitive function and to relieve AD symptoms such as decline of cognition, dementia, and depression. So, searching for new treatments for protection and curing AD from plant sources is an important era of research, as all available treatments for AD have limited effectiveness (18). Turmeric tubers (*Curcuma longa* L., Family Zingiberaceae) contain curcumin, a polyphenolic yellow substance represented by more than 60% of the active compounds of turmeric (19). Turmeric is among the most widely consumed dietary supplements globally (20). Numerous studies have been conducted on curcumin, a compound that exhibits a diverse range of effects, including anti-inflammatory, anti-oxidant, and anticancer properties (21). Zhang *et al*. (22) reported that turmeric (5 mg/day) improves neurocognitive functions of AD disease and reduces amyloid plaque and tau phosphorylation. Black pepper (*Piper nigrum* L., Family Piperacea) is used in traditional medicine due to its active compounds such as piperidine, α-pinene, and β-pinene (23). Black pepper possesses many medicinal properties such as anti-oxidant, anti-inflammatory, anticancer, and anti-atherosclerotic (24-26). In a sporadic AD mouse model, Piperine (2.5-10 mg/kg body weight) reduced cognitive impairment through neurotransmission restoration, neuroinflammation reduction, and oxidative stress amelioration (27). Wheat germ oil is a rich source of unsaturated fatty acids, tocopherols, policosanol, and phytosterols (28-30). Feltre *et al*. (28) reported that tocopherols showed several health benefits, such as anti-oxidant activity, retarded aging, delayed progression of degenerative disease, and improved dyslipidemia. Previous studies reported the therapeutic activities of wheat germ oil, such as anti-inflammatory, cardio-protective, hypolipidemic, and anti-oxidant (29-31).

For several decades, herbs and spices have been used as alternative therapy for many chronic diseases. To improve these therapeutic effects, multiple herb combinations can be used to enhance and optimize the synergistic effect of their different phytochemicals. So, the present research was a trial for optimizing the utility of dietary supplements containing a mixture of wheat germ oil and black pepper ethanol extract/or turmeric ethanol extract. Most previous studies included only one of these plants used in the present research. Thus, the present research aims to prepare dietary supplements and evaluate their impact on cholinergic neurotransmission, GSK3, APP, and Akt gene expression, oxidative stress, and inflammatory markers in a model of AD induced in rats. Also, a molecular docking study was applied to some phytochemicals in the dietary supplements to study their impact on AChE and Aβ.

## Materials and Methods

***Materials***

***Plant materials and chemicals***

Turmeric root (*Curcuma longa*), black pepper seeds (*Piper nigrum*), and wheat germ were purchased from local markets in Giza, Egypt. The chemicals and pure reagents were procured from Sigma Chemical Companies (Sigma-Aldrich, St. Louis, MO, USA). 

***Animals and diets***

Male rats of Sprague Dawley with a mean body weight of 218.4 g were used in the current investigation. Animals were purchased from the National Research Centre Animal House. During the study, the animals were given clean water and fed a balanced diet, as described previously by Mohamed *et al*. (32) *ad libitum* throughout the duration of the experiment. All animals were housed individually in cages. The animal procedures were done according to our institutional research and ethics committee and in line with the ethical guidelines for animal care and use for scientific purposes developed by the National Research Centre, Egypt (19176).

***Methods***

***Preparation of wheat germ oil, crude ethanol extract of black pepper, and turmeric***

Wheat germ oil was extracted by n-hexane according to the method previously described by Mohamed *et al*. (33). After evaporation of the solvent, wheat germ oil was frozen until use. To prepare crude ethanol extract, the air-dried powdered turmeric root and black pepper seeds were successively extracted separately with ethanol in the Soxhlet apparatus until exhausted. The solvent was totally eliminated through evaporation under vacuum, maintaining a temperature below 40 ^°^C. The crude extract of turmeric root and the crude ethanol extract of black pepper were stored in deep-freeze until utilized. 

***Preparation of dietary supplements***

Three dietary supplements (I, II, and III) were formulated. The dietary supplement I consisted of a mixture of black pepper crude ethanol extract and wheat germ oil in a 1:1 ratio. Dietary supplement II consisted of a 1:1 ratio of crude ethanol extract of turmeric and wheat germ oil. In contrast, dietary supplement III was composed of a 1:1:1 ratio mixture of crude ethanol extract of black pepper, crude ethanol extract of turmeric, and wheat germ oil. All dietary supplements were prepared as oil-in-water emulsions, with Tween 80 serving as the surfactant. First, the surfactant was dispersed in each oil phase separately using mechanical steering for 20 min. Then, each oil-surfactant mixture was added drop-wise to the aqueous phase (distilled water) to spontaneously form a coarse milky white emulsion. The three dietary supplements emulsions were further homogenized to reduce the particle size of the dispersed oil droplets by using a high-speed rotor-stator homogenizer (WiseTis HG-15D, Wise Laboratory instruments, Korea) at 5000 rpm for 5 min. The emulsions were stored at a temperature of 4 ^°^C for one week throughout the duration of the experiment.

***Extraction and determination of total phenolic compounds in the prepared dietary supplements***

The extraction of phenolic compounds from the prepared dietary supplements was conducted using a mixture consisting of methanol (80%) and hexane (30). The content of total phenolic compounds in dietary supplements was measured using the Folin-Ciocalteu method (34)**,** and the absorbance was measured by a spectrophotometer at 765 nm. The total phenolic content was reported as gallic acid equivalents (GAE) in mg/g of extract. 

***High-performance liquid chromatography (HPLC)*** ***analysis of phenolic compounds profile of the prepared dietary supplements***

Phenolic compounds extracted from the prepared dietary supplements (100 mg) were dissolved in 1 ml methanol (HPLC grade) and filtrated through a 0.2 μm filter sterilized membrane before the injection. Samples were determined in triplicate. HPLC analysis was carried out using an Agilent 1260 series. The separation was carried out using the Zorbax Eclipse Plus C18 column (4.6 mm x250 mm i.d., 5 μm). The mobile phase consisted of water (A) and 0.05% trifluoroacetic acid in acetonitrile (B) at a flow rate of 0.9 ml/min. The mobile phase was programmed consecutively in a linear gradient as follows: 0 min (82% A); 0–1 min (82% A); 1-11 min (75% A); 11-18 min (60% A); 18-24 min (82% A). The multi-wavelength detector was monitored at 280 nm. The injection volume was 5 μl from each sample solution in the Agilent HPLC, and the column temperature was maintained at 40 ^°^C. Retention times of the identified compounds were recorded. The concentration of each compound in the samples was estimated by comparing the peak area of the samples with the relative standards. The standards used were gallic acid, chlorogenic acid, catechin, methyl gallate, coffeic acid, syringic acid, pyrocatechol, rutin, coumaric acid, vanillin, ferulic acid, naringenin, rosmarinic acid, daidzein, querectin, cinnamic acid, kaempferol and hesperetin. 

***Determination of the anti-oxidant activity of the prepared dietary supplements***

The DPPH (2,2-diphenyl-1-picrylhydrazyl) assay (35) was used to measure the anti-oxidant activity of all dietary supplements. The following equation was used to determine the percent DPPH scavenging effect: The percent of DPPH scavenging effect=(A0-A1/A0)×100. Where A0 is the control absorbance, A1 is the standard, and test samples absorbance. 

***Determination of fatty acids and phytosterols in the prepared dietary supplements***

GLC analysis of fatty acid profiles and phytosterols was performed on the prepared dietary supplements fatty acid methyl esters and phytosterols, which were prepared according to AOAC (30, 36) procedures. The same conditions used by Mohamed *et al*. (37) were applied to identify and evaluate methyl ester fatty acids and phytosterols.

***Molecular docking of bioactive compounds of*** ***the*** ***prepared dietary supplements***

A set of the major phytochemicals present and determined in the prepared dietary supplements were retrieved from the PubChem database (38). The retrieved compounds were minimized using the Avogadro molecular modeling software (version 1.2.0) and the MMFF94 force field (39). The target proteins, acetylcholinesterase (AChE; UniProt ID: P22303) and amyloid-β (UniProt ID: P05067) were retrieved from the UniProt database (40). The protein structures were prepared using AutoDock Tools 1.5.7 (41), including adding hydrogen atoms, removing water molecules, and assignment of partial charges. Molecular docking was performed using AutoDock Vina (42). The prepared phytochemicals and proteins were used as inputs for the docking process. The docking results were visualized using the BIOVIA software, version 2020 (43). 

***Experimental model of Alzheimer’s disease***

After a week of adaptation, the animals were divided into five groups (n=6 each). Group one was the control group, which received the standard diet. The rats in group two were administered daily intraperitoneal injections of 150 mg/kg body weight of D-galactose and 10 mg/kg body weight of AlCl_3_ to induce AD in rats, following the protocol described by Bilgic *et al*. (44) and Mohamed *et al*. (32). Rats from groups three, four, and five were subjected to daily intraperitoneal injections of an identical D-galactose and aluminum chloride dose. Additionally, they were orally administered dietary supplements I, II, and III at 200 mg/kg of body weight per day. All rats were fed a balanced diet for the duration of the experiment, which lasted three weeks. Weekly weight and food intake records were also taken. A feed efficiency ratio, body weight gain, and total food intake were estimated at the end of the study. Following overnight fasting, the rats were subjected to anesthesia via peritoneal injection of 6.6 mg/kg of Ketamine and 0.3 mg/kg of Xylazine. Blood samples were collected. Plasma was detached from all blood samples for estimation of butyrylcholinesterase (BuChE)(45) and acetylcholinesterase (AChE)(SUNLOG, Cat No. SL002Ra, ELISA kit). Brain samples were dissected to determine markers of oxidative stress and inflammation, as well as to conduct gene expression analysis after the decapitation of animals.

***Determination of inflammatory and oxidative stress markers in rat brain homogenate***

The brains of the rats were collected, rinsed in cold saline, and then homogenized in phosphate buffer with a pH of 7.4. The homogenates were centrifuged for 10 min at 4 ^°^C and 4000 rpm. The supernatant was utilized to assess catalase activity (46), malondialdehyde (MDA) levels (47), and glutathione peroxidase (GPx) activity (SUNLOG, Cat No. SL1033Ra, ELISA kit) as indicators of oxidative stress. The inflammatory marker, tumor necrosis factor-α (TNF-α), was assessed using the SUNLOG ELISA kit (Cat No.SL0722Ra).

***Hippocampal gene expression of GSK3, APP, and Akt of different experimental groups***

The extraction of total RNA from the hippocampus tissue of rats was performed using the PureLink® RNA Mini Kit (ambion® Life technologies TM) following the instructions provided by the manufacturer. The concentrations and purity of RNA were assessed using a NanoDrop spectrophotometer. The complementary DNA (cDNA) was generated using 1.5 µg of total RNA in a 20 µl reaction utilizing the RevertAid first strand cDNA synthesis kit (Thermo Fisher® invitrogenTM), following the instructions provided by the manufacturer. 

RT-PCR was carried out using a Rotor-Gene^®^ MDx instrument. The RT-PCR reaction mixture consisted of a 25 µl volume. It comprised 1.0 µl of template complementary DNA (cDNA), 1× concentration of the EvaGreen® PCR master mix (HOT FIREPol® EvaGreen® qPCR Mix Plus, Solis BioDyneTM), and 0.2 µM concentration of the primer pairs. The primer pairs sequences utilized for the analysis of gene expression in glycogen synthase kinase-3 (GSK-3), amyloid precursor protein (APP), and serine/threonine protein kinase B (Akt) are provided in Table 1. 

The experimental protocol was as follows: 50 ^°^C for 2 min, then 95 ^°^C for 10 min, 45 cycles of 20 sec at 95 ^°^C, 30 sec at 56/60 ^°^C, and 30 sec at 72 ^°^C. Additionally, a melting curve program was employed, spanning a temperature range of 60-95 ^°^C. The relative expressions of the target genes were calculated using the 2-∆∆CT method, as described by Livak and Schmitting (48). The expression levels of the target genes were normalized to the expression levels of the housekeeping gene GAPDH.

***Statistical analysis***

The SPSS statistical program was used to analyze the data using one-way ANOVA and the Tukey multiple comparison test. Statistical significance was determined at a significance level of *P*≤0.05. 

## Results

***Total phenolic compounds and anti-oxidant activity of the prepared dietary supplements***

**Figure 1:**
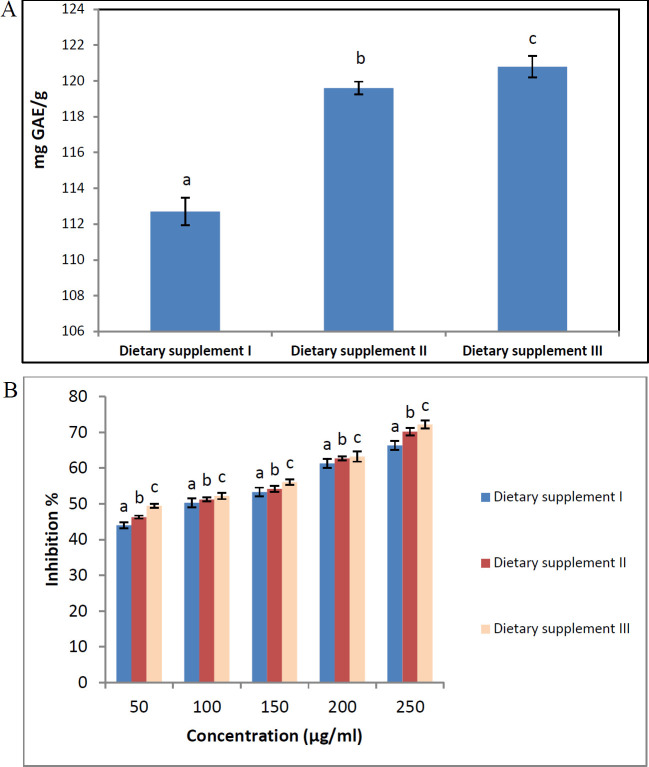
Prepared dietary supplements total phenolic compounds content and free radical scavenger activity

Figure 1A shows that total phenolic content in dietary supplements I, II, and III was present at 112.7, 119.6, and 120.8 mg GAE/g dietary supplements. Dietary supplement III exhibited the highest level of phenolic compounds, followed by dietary supplement II. Dietary supplement I exhibited the lowest level of total phenolic compounds. The anti-oxidant activity of dietary supplements I, II, and III was determined using DPPH radical (Figure 1B). Dietary supplement III exhibited the highest activity in scavenging DPPH radicals, followed by dietary supplement II in all the concentrations studied (50, 100, 150, 200, and 250 µg/ml). 

***HPLC phenolic profile of the prepared dietary supplements***

Phenolic compound profiles of the studied dietary supplements are presented in Table 2. The results of HPLC analysis of the studied dietary supplements revealed the presence of 16 phenolic compounds in dietary supplement I. However, 17 phenolic compounds were identified in dietary supplement II and 18 in dietary supplement III. Kaempferol was the major identified compound in dietary supplement II (8311.96 µg/g) and dietary supplement III (4160.4 µg/g), while ellagic acid was the major compound in dietary supplement I. Querectin (4 µg/g) was the minor compound identified in the dietary supplement I. Coumaric acid (8.2 µg/g) was the minor compound identified in the dietary supplement II, while rutin (4.9 µg/g) was the minor compound identified in the dietary supplement III. 

***Fatty acids profile and phytosterols of the studied dietary supplement***

Table 3 shows the prepared dietary supplements’ fatty acid profile and phytosterol content. In the present study, wheat germ oil was the main source of fat in all the prepared dietary supplements. So, all the investigated dietary supplements’ fatty acids profile and phytosterols content were identical. In the present study, the fatty acids profile of the prepared dietary supplements showed that linoleic acid (C 18:2, ω 6) was the highest unsaturated fatty acid present in wheat germ oil (54.4%), followed by oleic acid (C 18:1)(15.5%). Pllamitic acid (C 16:0)(16.2%) was the highest saturated fatty acid in the prepared dietary supplements. Total saturated fatty acids were present by 16.74%, while total unsaturated fatty acids were present by 70%. Total phytosterols were present in the prepared dietary supplements by 3.84%. β-Sitosterol (2.9%) was the major phytosterol present in the prepared dietary supplements followed by campesterol (0.64%). Stigmasterol (0.3%) was the lowest phytosterol present in the prepared dietary supplements. 

***In silico molecular docking of bioactive compounds of the prepared dietary supplements***

Table 4 presented the binding affinities (ΔG in kcal/mol) of selected phytochemicals determined in the prepared dietary supplements against the acetylcholinesterase (AChE) and amyloid-β (Aβ). The data revealed that rosmarinic and piperine exhibited the strongest binding affinities for AChE, while β-sitosterol showed the highest binding affinity for Amyloid-β. Several compounds, including ellagic acid, quercetin, curcumin, kaempferol, and hesperetin, display relatively high binding affinities for both AChE and Amyloid-β, suggesting their potential as dual-targeting agents for the treatment of neurodegenerative diseases like Alzheimer’s. The overall results highlight the promising therapeutic potential of these phytochemicals against AD. Rosmarinic acid and β-sitosterol showed the strongest binding affinities for AChE and Amyloid-β, respectively, among all phytochemicals measured in the prepared dietary supplements.

Table 5 provides the binding interactions between the phytochemical rosmarinic with the acetylcholinesterase (AChE) enzyme and the binding interactions between the phytochemical β-Sitosterol and the Amyloid-β protein. Rosmarinic exhibits a complex binding mode with AChE, as evidenced by the multiple hydrogen bond interactions with key amino acid residues, including ARG296, PHE295, TRP286, and TYR72. These hydrogen bonds likely contribute to the strong binding affinity of rosmarinic to the AChE active site. Additionally, rosmarinic engaged in favorable pi-pi stacking and T-shaped interactions with aromatic amino acid residues such as LEU289, TYR341, and TRP286, further stabilizing the ligand-enzyme complex. 

β-Sitosterol exhibited a hydrophobic binding mode with the Aβ protein, primarily engaging in alkyl and pi-alkyl interactions with several amino acid residues. These include MET458, HIS457, VAL454, PHE391, HIS388, and TYR217. The predominance of these hydrophobic interactions suggests that β-Sitosterol is well-accommodated within the hydrophobic pockets of the Aβ protein structure, potentially contributing to its high binding affinity. The unique binding modes and the involvement of different amino acid residues highlight the distinct binding characteristics of rosmarinic and β-Sitosterol, which may be crucial for their respective efficacies in modulating AChE activity and Aβ protein, respectively, and potentially impacting the treatment of AD. 

***Impact of dietary supplement administration on cholinergic neurotransmission***

**Figure 2:**
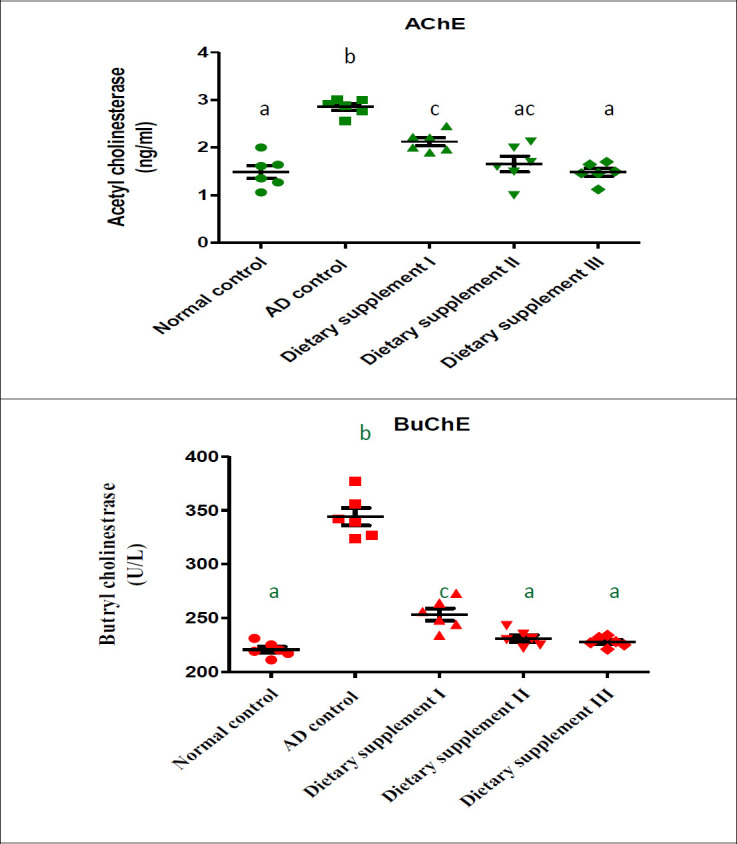
Effect of dietary supplement administration on plasma acetylcholinesterase (AChE) and butrylcholinesterase (BuChE) compared with normal and AD rats

The biochemical changes that occurred in the plasma and brain tissue of the numerous experimental groups are shown in Figure 2. Plasma levels of acetylcholinesterase and butrylcholinesterase showed significant elevation in the rats’ group of Alzheimer’s disease in comparison to the normal control group. Oral administration of different dietary supplements during injection with D-galactose and aluminum chloride reduced the elevation in plasma levels of acetylcholinesterase and butrylcholinesterase significantly compared with the rats’ group of Alzheimer’s disease. Dietary supplements II and III return plasma levels of butrylcholinesterase to normal levels, while dietary supplement III returns plasma levels of acetylcholinesterase. Rise in acetylcholinesterase, accompanied by an injection of D-galactose and aluminum chloride, is a valued marker of the rat model for AD. The present results indicated that the studied dietary supplements were very effective in improving the nervous system enzymes, which elevated significantly due to the injection of D-galactose and aluminum chloride. 

***Impact of dietary supplement administration on inflammatory and oxidative stress markers in rats’ brain***

Oxidative stress parameters of brain tissue of different experimental groups are illustrated in Table 6. Anti-oxidant enzymes catalase and glutathione peroxidase were reduced significantly in rats with AD. Rats given a daily oral dose of different dietary supplements showed significant catalase and glutathione peroxidase elevations as indicators of anti-oxidant status. Dietary supplement III was the most promising one. Malondialdehyde (MDA) as an indicator of lipid peroxidation in the brain tissue showed a significant increase in rats’ group of Alzheimer’s disease compared with all groups. The reduction of anti-oxidant enzymes and the elevation of MDA are indicators of the elevation of oxidative stress in rats with AD. Tumor necrosis factor-α as an inflammatory marker showed a significant elevation in the rats’ group of Alzheimer’s disease in comparison with all studied groups. All rats administered with dietary supplements I, II, or III indicate a significant reduction in TNF-α as an inflammatory marker and MDA as a lipid peroxidation marker associated with elevated anti-oxidant enzymes (catalase and glutathione peroxidase) to different degrees.

Table 7 presents the nutritional parameters investigated in the numerous experimental groups. The group of rats with AD exhibited a significant decrease in body weight gain and food intake compared to the group of normal rats. The administration of all dietary supplements resulted in enhanced body weight gain and food intake in the group of rats with Alzheimer’s disease. 

***Impact of dietary supplement administration on hippocampal gene expression of GSK3β, APP, and Akt***

**Figure 3:**
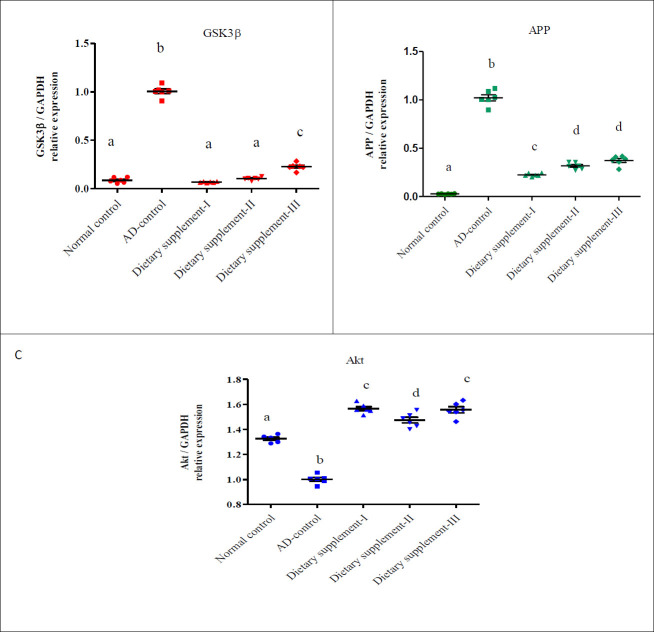
Effect of the prepared dietary supplements on the relative gene expression of GSK3β, APP, and Akt in the hippocampus of AD rats

GSK3β, APP, and Akt gene expression were detected using real-time-PCR in hippocampal tissue (Figure 3). The mRNA levels of GSK3β and APP were significantly increased in the Alzheimer’s disease rats group compared with normal rats. Treatments with dietary supplements I, II, or III significantly down-regulated the expression of GSK3β (Figure 3A) by 93%, 90%, and 80%, respectively. Dietary supplements I, II, and III significantly down-regulated APP expression by 78%, 69%, and 65%, respectively (Figure 3B). Akt gene expression (Figure 3C) was significantly reduced in the Alzheimer’s disease rats group compared with normal rats. However, the gene expression of Akt was significantly up-regulated by treatments with dietary supplement I, dietary supplement II, and dietary supplement III, respectively. 

## Discussion

AD is a neurogenerative disorder characterized by intraneuronal neurofibrillary tangles, extracellular amyloid plaques, synaptic loss, oxidative stress, neuropil threads, and neural loss (6). Several hypotheses attempt to describe AD pathogenesis, such as amyloid hypothesis, cholinergic hypothesis, tau hypothesis, oxidative stress, and neuroinflammation (4-6). Appropriate animal models are an important strategy to understand AD pathophysiology at the cellular, molecular, and behavioral levels and to develop new therapeutic agents. Aluminum exhibits a neuro/cholinotoxin-like effect that affects neuronal structure (52), permeability of the blood-brain barrier (BBB), and cholinergic/noradrenergic neurotransmission (53, 54). The BBB is altered by aluminum chloride exposure, which also affects axonal transports, causes inflammatory reactions, abnormal synaptic structural changes, and profound memory loss (54). The potential neurotoxicity effect of aluminum was also confirmed on experimental animal models (55-57, 32), demonstrating that chronic exposure to aluminum ions results in neurologic symptoms resembling progressive neurodegeneration in the spinal cord, cerebral cortex, and hippocampus (32, 54). It was reported previously that a combination of AlCl_3_ and Dgalactose was effective as an animal model, which induces different pathological changes mimicking AD in humans, such as neuronal loss, dementia, and elevated acetylcholinesterase activity (32, 58). The current study explored the influences of three prepared dietary supplements on cholinergic function and amyloid-β through evaluation of their impact on cholinergic neurotransmission (AChE and BuChE), gene expression of GSK3β, APP, and Akt, oxidative stress and inflammatory markers in Alzheimer’s disease rats’ models induced by injection of D-galactose and aluminum chloride as well as an *in silico* molecular docking study. 

Phytochemicals from plants are considered a good strategy for protecting and/or treating AD as they play an important role as complementary or alternative therapies for AD. Plants’ phytochemicals proved anti-oxidant and anti-inflammatory activities and exhibited therapeutic impact on neurodegeneration disorders (59). In the present study, three dietary supplements were prepared and evaluated to prevent AD in rat models. Dietary supplement I contains crude ethanol extract of black pepper and wheat germ oil (a ratio of 1:1), and dietary supplement II contains crude ethanol extract of turmeric and wheat germ oil (a ratio of 1:1). In contrast, dietary supplement III contains crude ethanol extract of turmeric, crude ethanol extract of black pepper and wheat germ oil in a ratio 1:1:1. *in silico* molecular docking study of the main bioactive compounds of the three prepared dietary supplements in the current research revealed that rosmarinic, piperine, quercetin and curcumin exhibited the strongest binding affinities for AChE. These high binding affinities can be attributed to the ability of these phytochemicals to form multiple hydrogen bonds and favorable pi-stacking interactions within the AChE active site. β-Sitosterol showed the highest binding affinity for amyloid-β. The strong binding of β-Sitosterol can be attributed to its ability to engage in alkyl and pi-alkyl interactions with the hydrophobic pockets within the amyloid-β structure. Hydrogen bonding, pi-stacking, and hydrophobic interactions are crucial for binding, specifically for optimizing binding affinity and enhancing the stability of drug-protein complexes (60). The distinct binding modes and the involvement of different types of interactions, such as hydrogen bonding, pi-stacking, and hydrophobic interactions, highlight the unique molecular recognition mechanisms of currently studied compounds in the three dietary supplements, which may be crucial for their respective efficacies in modulating the activities of AChE and amyloid-β, ultimately impacting the treatment of AD.

The results revealed that all the studied dietary supplements effectively reduced the elevation of acetylcholinesterase and butrylcholinesterase in plasma, following reduced oxidative stress and inflammation markers in the brain tissues. The protective effect of the studied dietary supplements may be attributed to the presence of phenolic compounds. Likewise, it may be attributed to the presence of polyunsaturated fatty acids and phytosterols. In the current research, the prepared dietary supplements contain crude ethanol extract from turmeric and/or crude ethanol extract from black pepper and wheat germ oil. It was reported previously that curcumin (60% of the active compounds of turmeric) is the major polyphenol compound present in turmeric (19). The curcumin structure includes reactive functional groups, such as diketone and phenol, crucial in scavenging reactive oxygen species (ROS) and working like anti-oxidant enzymes (61). Previously, curcumin was shown to have neuroprotection activity in AD models in rats (62-64). Black pepper possesses many medicinal activities such as anti-inflammatory, anti-oxidant, and anti-atherosclerotic (25-27) due to the presence of phytochemical compounds such as piperidine, α-pinene, and β-pinene (23). Piperine proved to have anti-inflammatory activity in rat models of carrageenan-induced rat paw edema and a croton oil-induced granuloma pouch (65). Ethanol extract from black pepper showed marked decreased cholinesterase levels and amyloidal plaque formation in rat brains (66, 67). Rosmarinic acid, present in the three dietary supplements, showed high and strong binding affinities for AChE and amyloid-β, as approved in our research by the molecular docking *in silico* study. Yamamoto *et al*. (68) reported that rosmarinic acid reduced inflammation in rats’ brains and suppressed tau’s phosphorylation via a down-regulating JNK signaling pathway. Also, rosmarinic acid suppresses the accumulation of amyloid-β in mice (69). 

Wheat germ oil was used in combination with turmeric extract and crude ethanol extract of black pepper to enhance the bioavailability of phytochemicals present in turmeric and black pepper, especially curcumin and piperine. Curcumin and piperine are lipophilic compounds, so adding wheat germ oil enhances their activities to cross the blood-brain barrier, inhibit lipid peroxidation, and elevate their activities as anti-oxidants (70,71). Wheat germ-oil is one of the richest sources of phytosterols (especially β-Sitosterol) and tocopherols. According to an *in vivo* study, β-Sitosterol exhibited an inhibition effect on enzymes involved in cholinesterase’s metabolism and acted as a free radical scavenger (72). β-Sitosterol revealed the strongest binding affinities for amyloid-β in our study. Hence, combining wheat germ oil with turmeric and/or black pepper crude ethanol extracts elevates their activities as anti-oxidants, anti-inflammatory, and in treating neurodegeneration (28-30, 33). 

Glycogen synthase kinase 3β (GSK3 β) is a Serine/Threonine protein kinase that has garnered significant interest due to its involvement in numerous pathways. GSK3β is constitutively active and abundantly expressed in the central nervous system (16). It has been suggested that GSK3β serves as a molecular bridge between tau and amyloid-β in the pathogenesis of AD. Amyloid-β activates GSK3β, which in turn phosphorylates tau (73). There has been a proposition indicating that GSK-3 may play a pivotal role in the process of epileptogenesis in AD through its interaction with the pathological features of AD, namely amyloid precursor protein (APP) and tau (74). In contrast, GSK3β plays a role in regulating APP metabolism and Aβ production and in promoting neuronal death triggered by Aβ (75, 76). Aβ and its precursor protein are considered the keystone of the pathogenesis of Alzheimer`s disease (77). Postmortem brains from AD patients had higher GSK3β levels than age-matched control samples (53,73). GSK3β activation has been demonstrated to promote the production of inflammatory markers such as TNF-α, IL-1, and IL-6 (78), as shown in the present study. GSK3β has been regarded as a crucial target in treating AD due to its high specificity in substrate recognition (79). Akt negatively regulates GSK3β activity, protecting cells against the effect of GSK3β (51). In the current study, dietary supplement-I, dietary supplement-II, and dietary supplement-III decreased the expression of GSK3β and APP and increased the expression of Akt, which resulted in a decrease in neurotoxicity (promoting neuronal survival or enhanced Alzheimer’s status). Thus, the modulation of the GSK3β /APP signaling pathway and Akt/GSK3β signaling pathway by natural dietary supplements is a promising target for an Alzheimer’s therapeutic approach. 

**Table 1:** Primers sequence used for real-time PCR analysis of gene expression of GSK3, APP, and Akt

Ref.	Annealing temperature	Sequences	Target genes
This study	60 °C	FW (5′-CGGGACCCAAATGTCAAAC-3′) RW(5′-CGTGACCAGTGTTGCTGAG-3′)	GSK3
[49]	56 °C	FW (5′-ACCCATCAGGGACCAAAACC3') RW(5′-GGCATCGCTTACAAACTCACC3')	APP
[50]	60 °C	FW (5′-ACTCATTCCAGACCCACGAC-3′) RW(5′- CCGGTACACCACGTTCTT-3′)	Akt
[51]	60 °C	FW (5′- GTATCGGACGCCTGGTTACC-3′) RW(5′- CGCTCCTGGAAGATGGTGATGG-3′)	GAPDH

A: total phenolic as mg GAE/g; B: anti-oxidant activity. The same letter means non-significant difference, while a different letter means significant difference at 0.05 probabilities. The data are expressed as mean values±standard error

**Table 2:** Phenolic compounds levels (µg/g) in the all prepared dietary supplements

Phenolic compounds	Dietary supplements I	Dietary supplements II	Dietary supplements III
Gallic acid	55.2±0.046	127.91±0.081	91.6±0.060
Chlorogenic acid	16.4±0.070	53.51±0.085	34.9±0.055
Catechin	-	27.3±0.065	13.59±0.035
Methyl gallate	93.59±0.060	16.1±0.061	54.8±0.050
Coffeic acid	43.84±0.056	54.5±0.060	49.2±0.056
Syringic acid	67.2±0.035	79.4±0.081	73.3±0.066
Rutin	9.79±0.075	-	4.89±0.055
Ellagic acid	315.5±0.066	14.8±0.031	165.11±0.046
Coumaric acid	8.2±0.042	351.9±0.055	180.11±0.051
Vanillin	46.4±0.055	553.2±0.057	299.82±0.053
Ferulic acid	-	523.8±0.070	261.89±0.046
Naringenin	10.0±0.051	14.2±0.046	12.16±0.051
Rosmarinic acid	129.66±0.0115	132.7±0.061	131.2±0.047
Daidzein	21.49±0.066	121.5±0.055	71.5±0.061
Querectin	280.29±0.060	4.05±0.050	142.1±0.057
Cinnamic acid	9.39±0.055	91.6±0.070	50.51±0.066
Kaempferol	8.74±0.056	8312±0.080	4160.4±0.065
Hesperetin	10.78±0.044	598.08±0.076	304.4±0.055

**Table 3:** Fatty acids and phytosterols levels in the all prepared dietary supplements

Fatty acids	Dietary supplements
Fatty acids as percentage of total fatty acids	
Palmitic C16:0	16.25±0.003
Stearic C18:0	0.57±0.03
Oleic, C18:1	15.77±0.252
Linoleic, C18:2	54.97±0.551
α-Linolenic, C18:3	6.67±0.416
Total identified saturated fatty acids	16.82±0.080
Total identified unsaturated fatty acids	77.4±1.153
Phytosterols (as percentage of total phytosterols)	
Campesterol	0.687±0.042
Stigmasterol	0.343±0.040
β-Sitosterol	3.067±0.153
Total phytosterols	4.097±0.224

**Table 4:** Binding affinity for docking experiment of phytochemicals with AChE and Amyloid-β

Compounds	Binding affinity (ΔG in kcal/mol)	
	AChE	Amyloid-β
Curcumin	-9.3	-7.7
Ellagic acid	-9.4	-8.0
Hesperetin	-8.5	-7.7
Kaempferol	-9.2	-7.6
Piperine	-10.3	-7.4
Quercetin	-9.7	-7.6
Rosmarinic	-10.5	-7.4
Linoleic	-6.6	-5.2
Oleic acid	-6.8	-4.5
β Sitosterol	-9.3	-8.4

**Table 5:** 3D and 2D for Rosmarinic with AChE and β-Sitosterol with Amyloid-β

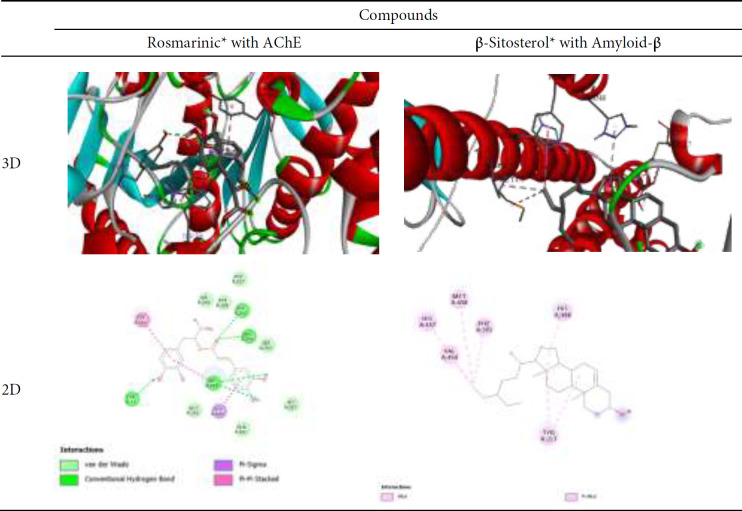

* Rosmarinic exhibits the strongest binding affinities for AChE among all phytochemicals measured in the prepared dietary supplements; β-Sitosterol exhibits the strongest binding affinities for Amyloid-β among all phytochemicals measured in the prepared dietary supplements

The same letter means non-significant difference, while a different letter means significant difference at 0.05 probabilities. The data are expressed as mean values ± standard error

**Table 6:** Effect of the prepared dietary supplements on the inflammatory and oxidative stress markers in brain tissue of the AD rats

Parameters	Normal control	Alzheimer’s disease control	Dietary supplement I	Dietary supplement II	Dietary supplement III
Oxidative stress markers					
Catalase (U/g)	0.768^a^±0.041	0.53^b^ ±0.015	0.582^b^±0.032	0.609^b^±0.013	0.612^b^±0.017
Gpx (U/mg)	43.5^a^±0.294	30.28^b^±0.553	35.19^c^±0.942	37.54^d ^±0.64	40.43^e^±0.473
MDA (nmol/g tissue)	9.93^a^±0.219	24.19^b^±0.259	17.29^c^±1.34	11.81^a^±0.44	10.58^a^±0.345
Inflammatory marker					
TNF-α (ng/g tissue)	19.92^a^±0.305	36.69^b^±0.656	24.1^c^±1.06	21.07^a^±0.553	20.05^a^±0.297

In each raw, the same letter means non-significant difference, while a different letter means significant difference at 0.05 probabilities. The data are expressed as mean values±standard error

**Table 7:** Effect of the prepared dietary supplements on the nutritional parameters in AD rats

Parameters	Normal control	Alzheimer’s disease control	Dietary supplement I	Dietary supplement II	Dietary supplement III
Initial body weight (g)	218.5^a^±4.39	218.3^a^±2.23	218.3^a^±5.95	218.3^a^±5.26	218.3^a^±7.46
Final body weight (g)	251.3^a^±2.85	235.3^a^±4.14	241.7^a^±6.07	241.2^a^±4.72	243.8^a^±6.084
Body weight gain (g)	32.8^a^±5.62	17.0^b^±3.38	23.3^ab^±3.30	22.8^ab^±2.39	25.5^ab^±3.02
Total food intake (g)	347.17^a^±3.33	339.67^b^±4.81	351.3^ac^±2.20	346.3^a^±5.63	349.75^a^±6.88
Feed efficiency ratio	0.094^a^±0.015	0.052^a^±0.011	0.067^a^±0.009	0.065^a^±0.006	0.074^a^±0.008
Relative brain weight	0.518^a^±0.047	0.585^a^±0.039	0.564^a^±0.033	0.547^a^±0.027	0.504^a^±0.023

Similar letters mean non-significant differences within groups (*P≤*0.05) in the same raw

The mRNA expression of GSK3, APP, and AKT is normalized with the housekeeping gene (GAPDH); values are represented as Mean±SE, calculated as fold-change from AD control. The same letter in each column is not significantly different; different letters are significantly different at 0.05 probability levels

## Conclusion

The studied dietary supplements were considered promising for AD prevention. Dietary supplement III (mixture of crude ethanol extract of turmeric, crude ethanol extract of black pepper, and wheat germ oil) was the most promising in preventing oxidative stress and inflammation parameters. Molecular docking revealed that rosmarinic acid has the strongest binding affinities for AChE, while β-sitosterol was the most promising in preventing A-β aggregation. All dietary supplements enhanced cholinergic function and significantly down-regulated the expression of GSK3β and APP while significantly up-regulated the Akt gene expression. All dietary supplements may impact the existence of polyunsaturated fatty acids, phytosterols, phenolic compounds, and their anti-oxidant activity against free radicals. The most important finding in the present study was the positive synergistic effect of mixing wheat germ oil with crude ethanol extract of turmeric and/or crude ethanol extract of black pepper in the form of dietary supplements, which have potential activity on the prevention of AD. 

## Data Availability

All data generated or analyzed during this study are included in this published article.

## Declaration

We have not used any AI tools or technologies to prepare this manuscript.
